# Expression of GnT-III decreases chemoresistance *via* negatively regulating P-glycoprotein expression: Involvement of the TNFR2-NF-κB signaling pathway

**DOI:** 10.1016/j.jbc.2023.103051

**Published:** 2023-02-21

**Authors:** Wanli Song, Caixia Liang, Yuhan Sun, Sayaka Morii, Shin Yomogida, Tomoya Isaji, Tomohiko Fukuda, Qinglei Hang, Akiyoshi Hara, Miyako Nakano, Jianguo Gu

**Affiliations:** 1 Division of Regulatory Glycobiology, Institute of Molecular Biomembrane and Glycobiology, Tohoku Medical and Pharmaceutical University, Sendai, Miyagi, Japan; 2 Graduate School of Integrated Sciences for Life, Hiroshima University, Sendai, Miyagi, Japan; 3 Division of Clinical Pharmacotherapeutics, Tohoku Medical and Pharmaceutical University, Sendai, Miyagi Japan

**Keywords:** bisecting GlcNAc, chemoresistance, *N*-glycosylation, GnT-III, P-gp

## Abstract

The phenomenon of multidrug resistance (MDR) is called chemoresistance with respect to the treatment of cancer, and it continues to be a major challenge. The role of *N*-glycosylation in chemoresistance, however, remains poorly understood. Here, we established a traditional model for adriamycin resistance in K562 cells, which are also known as K562/adriamycin-resistant (ADR) cells. Lectin blot, mass spectrometry, and RT-PCR analysis showed that the expression levels of *N*-acetylglucosaminyltransferase III (GnT-III) mRNA and its products, bisected *N*-glycans, are significantly decreased in K562/ADR cells, compared with the levels in parent K562 cells. By contrast, the expression levels of both P-glycoprotein (P-gp) and its intracellular key regulator, NF-κB signaling, are significantly increased in K562/ADR cells. These upregulations were sufficiently suppressed by the overexpression of GnT-III in K562/ADR cells. We found that the expression of GnT-III consistently decreased chemoresistance for doxorubicin and dasatinib, as well as activation of the NF-κB pathway by tumor necrosis factor (TNF) α, which binds to two structurally distinct glycoproteins, TNF receptor 1 (TNFR1) and TNF receptor 2 (TNFR2), on the cell surface. Interestingly, our immunoprecipitation analysis revealed that only TNFR2, but not TNFR1, contains bisected *N*-glycans. The lack of GnT-III strongly induced TNFR2’s autotrimerization without ligand stimulation, which was rescued by the overexpression of GnT-III in K562/ADR cells. Furthermore, the deficiency of TNFR2 suppressed P-gp expression while it increased GnT-III expression. Taken together, these results clearly show that GnT-III negatively regulates chemoresistance *via* the suppression of P-gp expression, which is regulated by the TNFR2-NF/κB signaling pathway.

Chronic myeloid leukemia (CML) is a myeloproliferative malignancy that arises from hematopoietic stem cells (1), and it accounts for 15% of adult leukemias (2). CML classically progresses through three phases (chronic phase, accelerated phase, and blast phase) and becomes more resistant to treatment in each successive phase (3). Imatinib is a tyrosine kinase inhibitor of BCR-ABL1 that has become one of the most successful drugs yet approved for therapeutic use (4, 5). Its broad application, however, has spurred debate regarding the efficacy of both imatinib and later generations of tyrosine kinase inhibitor, and this opposition is due mainly to the acquisition of drug resistance (6). Therefore, overcoming multidrug resistance (MDR) is a big challenge for the successful treatment of CML or other cancers.

The emergence of the MDR phenotypes is primarily involved in the overexpression of MDR-associated genes such as ATP-binding cassette (ABC) subfamily B1 genes (P-gp/MDR1/ABCB1), ABCC1 (MRP1), and ABCG2 (BCRP1). Classic MDR is caused by the overexpression of P-glycoprotein (P-gp) (7). P-gp is the first efflux pump to be discovered that reduces the cellular toxicities of chemotherapeutic agents by lowering the intracellular drug accumulation of taxanes (paclitaxel), vinca alkaloids (vinblastine), and anthracyclines (daunorubicin) (8, 9, 10). Thus, to reverse MDR by inhibiting P-gp should re-sensitize tumor cells to antineoplastic agents and allow the successful treatment of multidrug-resistant cancer cells. Promoter analyses has revealed that the human P-gp gene promoter harbors NF-κB responsive elements, which bind with NF-κB to promote P-gp gene expression (11, 12) but that inhibition of the NF-κB signaling pathway decreases P-gp expression, which increases apoptotic cell death in response to daunomycin treatment in human colorectal adenocarcinoma cells (12). Tumor necrosis factor α (TNFα) is one of the most potent activators of NF-κB signaling. Several studies have shown that the TNFα/NF-κB signaling pathway mediates P-gp upregulation in both tumor and normal cells (13, 14, 15, 16, 17, 18). TNFα interacts with cells through specific receptors, TNF receptor 1 (TNFR1) or TNFR2, and triggers the downstream NF-κB pathway to regulate the expression of MDR (17, 19). TNFR1 can be modified by *N*-glycosylation (20, 21). When TNFR1 lacks *N*-glycosylation, its binding to TNFα is suppressed, which thereby represses TNFα-mediated NF-κB signaling (20).

*N*-Glycosylation is one of the most common forms of protein postmodification and is an important process in cells (22, 23). Aberrant *N*-glycosylation has been associated with cancer chemoresistance (24, 25, 26, 27). For example, elevating sialylated *N*-glycans or high mannose decreases the sensitivity of nonsmall lung cancer cells to cisplatin and contributes to the development of chemoresistance phenotypes (27). The overexpression of fucosyltransferase 4 (FUT4), FUT6, or FUT8 is responsible for MDR in human hepatocellular carcinoma *via* activation of the PI3K/Akt signaling pathway, which induces P-gp expression (28). The products of *N*-acetylglucosaminyltransferase V (GnT-V encoded by the Mgat5 gene), β1,6GlcNAc-branched *N*-glycans, have been associated with cancer growth, invasion, metastasis (29), and drug resistance (30, 31). The expression levels of GnT-V are known to be higher in the adriamycin-resistant (ADR) MCF-7 cells than in parental cells, and a knockdown of GnT-V has decreased the resistance to paclitaxel, doxorubicin (DOX), and vincristine (32). The expression levels of *N*-acetylglucosaminyltransferase IV (GnT-IV encoded by the Mgat4 gene), which is involved in β1,4GlcNAc-branched *N*-glycans, are also induced in the cisplatin-resistant variants of A2780 cells, compared with wildtype (WT) cells (33).

*N*-Acetylglucosaminyltransferase III (GnT-III encoded by the Mgat3 gene) is another important glycosyltransferase that has received much recent attention for its involvement in the biology of tumors (34, 35). GnT-III plays a critical role in defining the ultimate structure of hybrid and complex *N*-glycans as the addition of a bisecting GlcNAc suppresses further processing and elongation of *N*-glycans to form branching structures that are catalyzed by GnT-IV and GnT-V, because they are not able to use the bisected oligosaccharide as a substrate (36). Thus, GnT-III is generally regarded as a key glycosyltransferase in *N*-glycan biosynthetic pathways (37). Previous studies have shown that the expression levels of GnT-III are decreased in several drug-resistant cell lines (38, 39). Moreover, a knockdown of GnT-III has increased the resistance to 5-fluorouracil in human hepatocarcinoma cells (31). Taken together, these studies strongly suggest that glycosylation may play a potential role in chemoresistance as cancer cells acquire tolerance. However, only a few studies have investigated the underlying mechanisms between chemoresistance and *N*-glycans.

In the present study, we established a traditional model of a human CML cell line, K562/ADR cells, and found that the expression levels of GnT-III and bisected *N*-glycans were significantly decreased in these cells, compared with the parental K562 cells. Furthermore, our studies have clearly shown that TNFR2 is modified when the GlcNAc of *N*-glycans are bisected, which negatively regulates trimerization of the TNFR2 and TNFα/NF-κB signaling pathways for P-gp expression and contributes to cancer chemoresistance.

## Results

### Bisected N-glycans are decreased in K562/ADR cells

**Figure 1:**
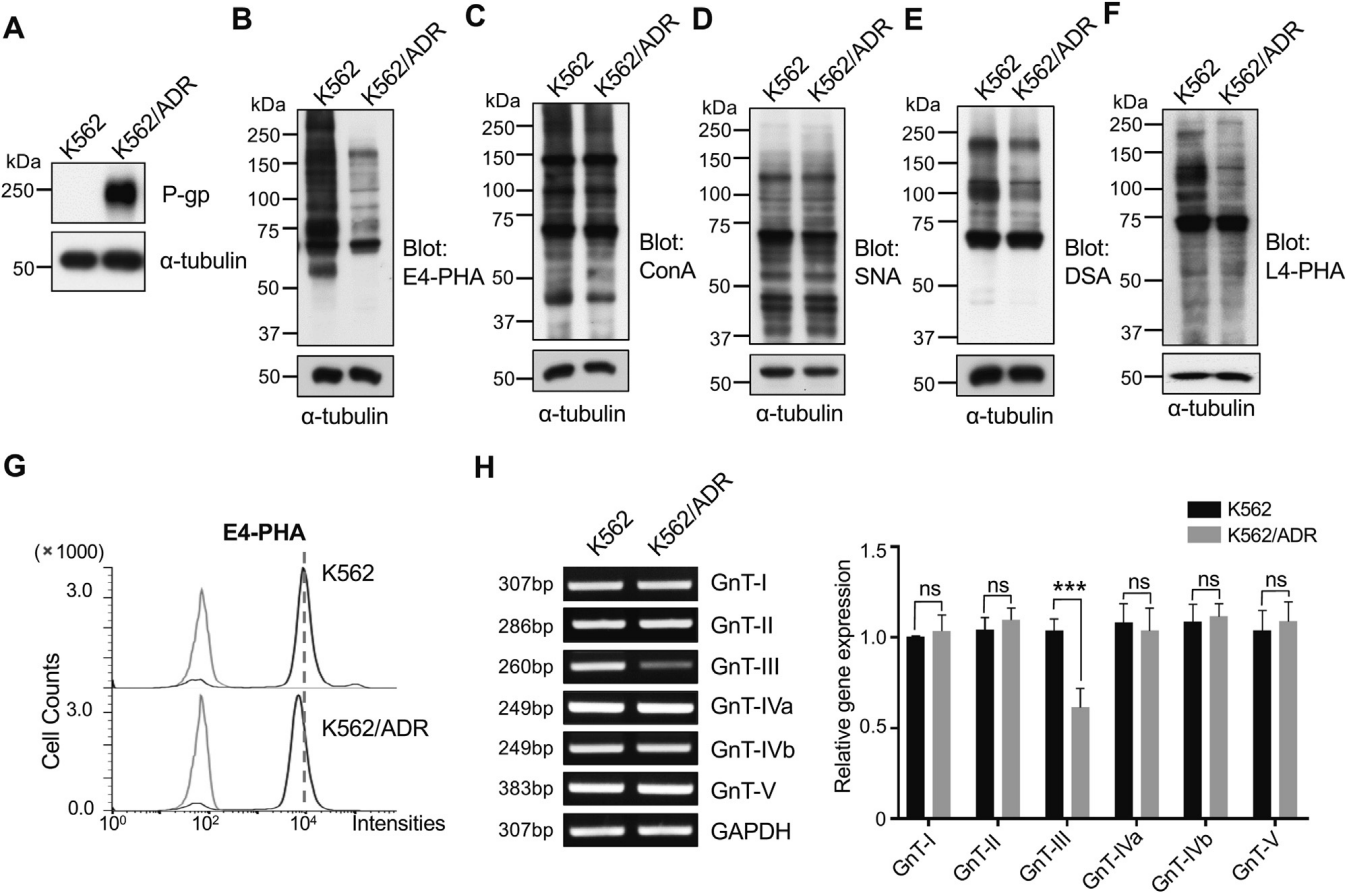
**Comparison of the expression levels of P-gp, glycans, and mRNA for the glycosyltransferases between K562 and K562/ADR cells.***A*, the expression levels of P-gp were analyzed by immunoblotting with an anti-P-gp antibody; α-tubulin served as a loading control. *B*–*F*, equal amounts of the cell lysates of K562 and K562/ADR cells were stained with E4-PHA, ConA, SNA, DSA, and L4-PHA lectin, respectively. α-tubulin was used as a loading control. *G*, the expression levels of bisecting GlcNAc on the cell surface were analyzed by flow cytometry using E4-PHA. *H*, the mRNA levels of several *N*-acetylglucosaminyltransferases, which are involved in GlcNAc branched *N*-glycan synthesis, were detected *via* RT-PCR; GAPDH was used as an internal control. The data were obtained from three independent experiments. All values are reported as the mean ± S.E.M (n = 3). ∗∗∗*p* < 0.001; n.s, no significance by two-way analysis of variance. ADR, adriamycin-resistant; P-gp, P-glycoprotein.

Elevated GnT-III expression and its products, bisected GlcNAc N-glycans, have been detected in patients with CML during blast crisis when compared with healthy controls or with patients with other hematological malignancies (40, 41). The K562/ADR cell is a traditional model for studying drug resistance in CML cells. First, we established the K562/ADR cell line using a stepwise increase in DOX concentrations, as described in the Experimental procedure section. As expected, the expression levels of P-gp were greatly increased in the K562/ADR cells compared with parent K562 cells (Fig. 1*A*).

To compare the glycan expression patterns of K562 and K562/ADR cells, we performed lectin blotting using four different lectins: E4-PHA, ConA, SNA, DSA, and L4-PHA. These lectins preferentially recognize the bisected *N*-glycans catalyzed by GnT-III, α-linked mannose including high mannose–type and hybrid-type *N*-glycans, α2,6 sialylation on a terminal branch, β1,4-GlcNAc-branched, and β1,6-GlcNAc-branched (tri-antennary) *N*-glycans, respectively. The reactivities of E4-PHA were dramatically decreased in K562/ADR cells compared with WT K562 cells, as validated *via* lectin blot (Fig. 1*B*) and flow cytometry (Fig. 1*G*). Further lectin blotting with ConA (Fig. 1*C*), SNA (Fig. 1*D*), DSA (Fig. 1*E*), or L4-PHA (Fig. 1*F*) showed no dramatic changes between the cells. It is worth noting that both DSA and L4-PHA lectin blots tended to decrease in K562/ADR cells compared with that in K562 cells, which seems to be contradictory to previous notion since reciprocal activity is known to exist between GnT-III and GnT-V or GnT-IV (36, 37). Thus far, it is difficult to explain the phenomena, which requires further study. Consistently, the results obtained from RT-PCR showed that the expression levels of GnT-III, as opposed to the levels indicated by other GnT-Ts, were decreased in K562/ADR cells compared with the levels in WT K562 cells (Fig. 1*H*).

**Figure 2:**
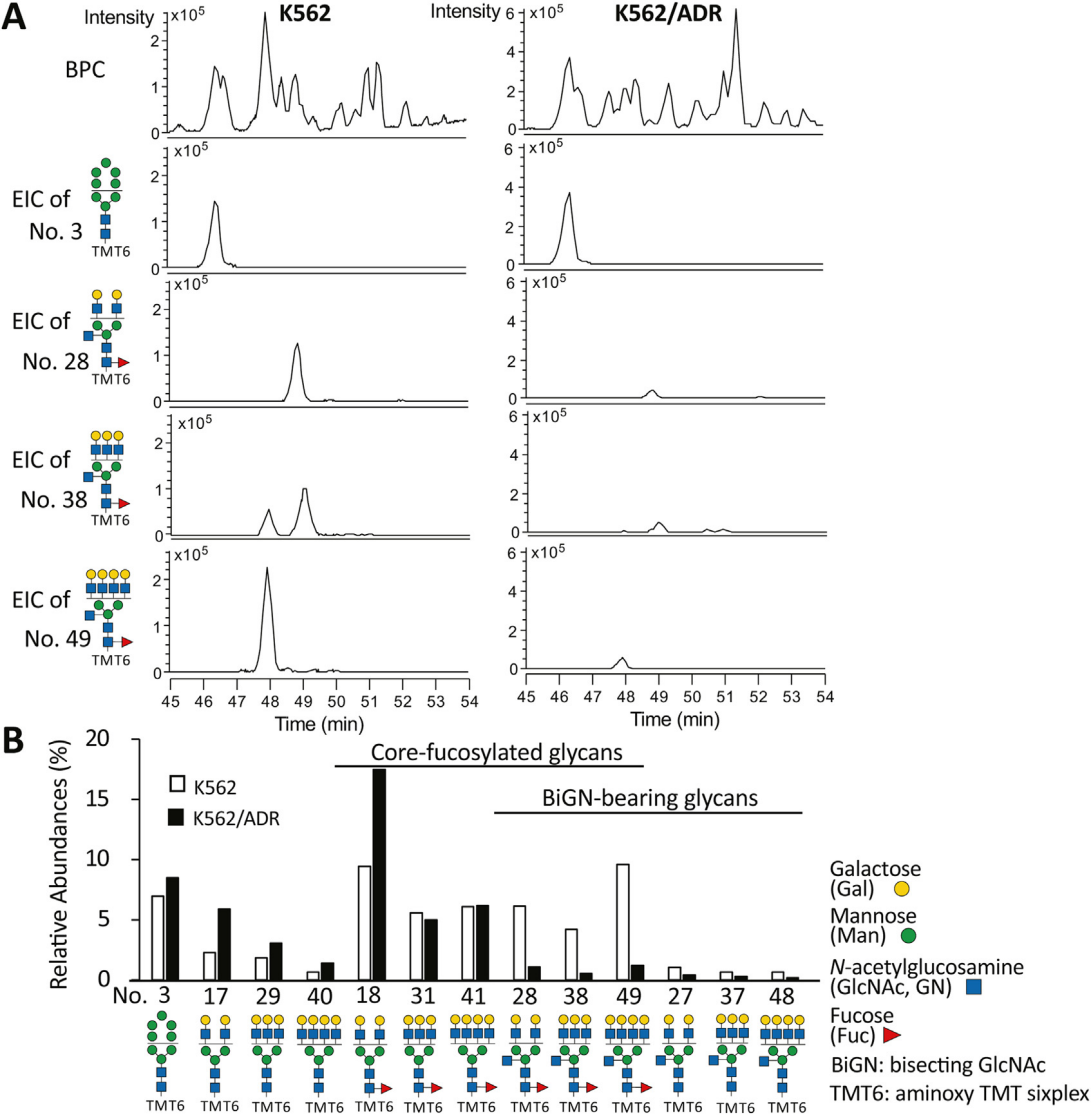
**LC-ESI MS analysis of *N*-glycans in K562 and K562/ADR cells.***A*, base peak chromatogram (BPC) and extracted ion chromatogram (EIC) of H5+C5T (structure No.3), H2N3F1+C5T (structure No.28), H3N4F1+C5T (structure No.38), and H4N5F1+C5T (structure No. 49) based on MS analysis of the *N*-glycans from K562 and K562/ADR cells. *B*, relative abundances (%) of a high mannose type of *N*-glycan (structure No.3), nonfucosylated, and nonbisecting GlcNAc-bearing glycans (structure No.17, 29, 40), core-fucosylated, and nonbisecting GlcNAc-bearing glycans (structure No.18, 31, 41), core-fucosylated and bisecting GlcNAc-bearing glycans (structure No.28, 38, 49), and nonfucosylated and bisecting GlcNAc-bearing glycans (structure No.27, 37, 48) of basal structures H2N2+C5T, H3N3+C5T, and H4N4+C5T. The numerical values used to create this graph are shown in Table S1. ADR, adriamycin-resistant; C5T, core 5 monosaccharides labeled with TMT6 (3 Mannoses + 2 *N*-acetylglucosamines + TMT6); F, Fucose; H, Hexose; N, HexNAc, TMT6, aminoxy Tandem Mass Tag sixplex.

Furthermore, to confirm the changes in the *N*-glycans shown in Figure 1, we isolated *N*-glycans from cell membrane proteins and desialylated them. The glycans were then labeled with aminoxy Tandem Mass Tag sixplex (TMT6) and analyzed *via* LC-ESI MS. Retention time, observed/theoretical mass, charge state, intensity, and composition of deduced structure of all observed *N*-glycans were summarized in the result file where have been deposited in GlycoPOST. As shown in Figure 2, *N*-glycan structures with bisecting GlcNAc were greatly reduced in K562/ADR cells, compared with the K562 cells, which is consistent with the results shown by lectin blots. These data strongly suggest that the expression levels of the bisected *N*-glycans are downregulated in the acquisition of drug resistance of leukemia cells.

### Overexpression of GnT-III suppressed P-gp in K562/ADR cells

**Figure 3:**
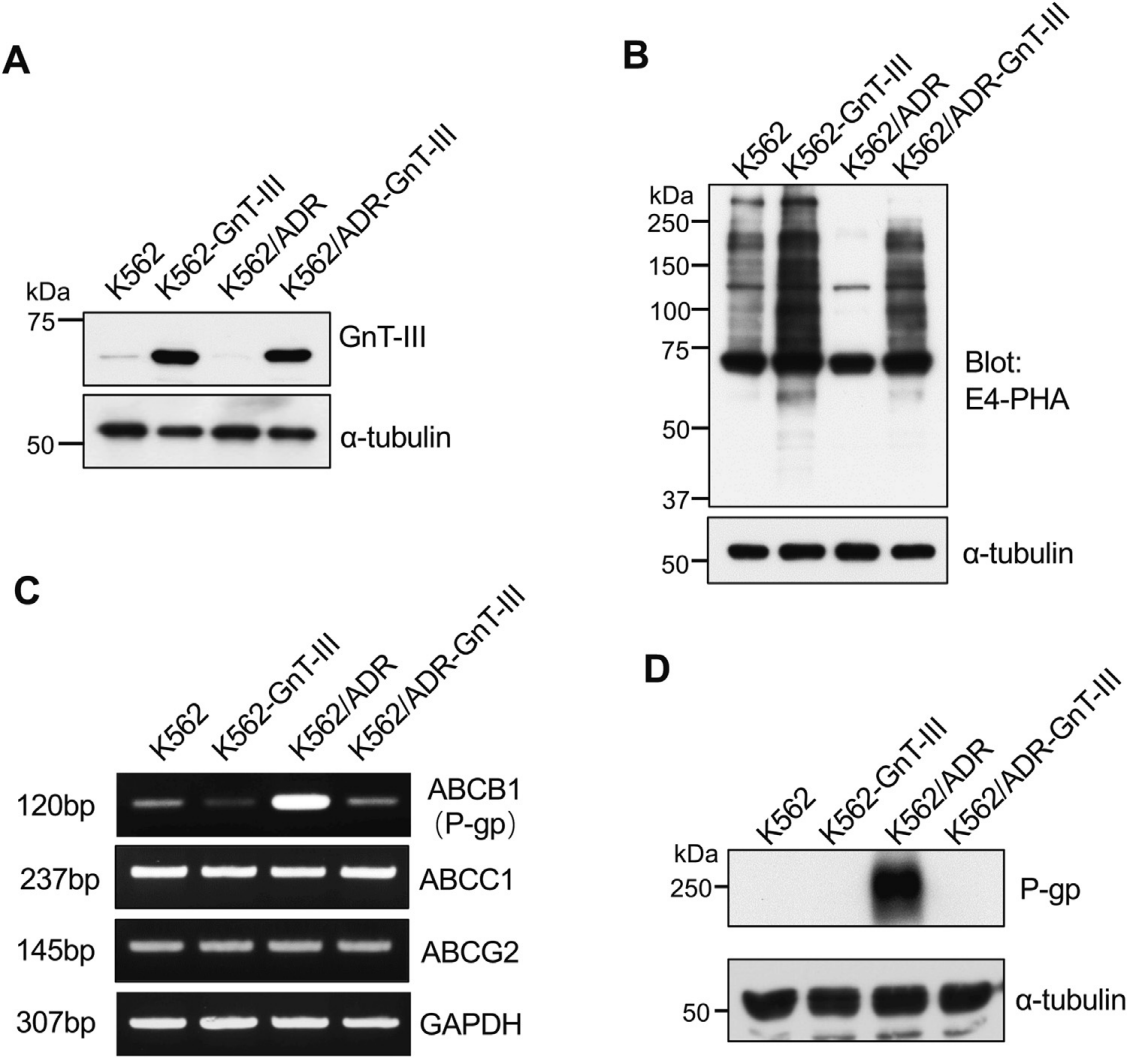
**Effects of GnT-III on P-gp expression.** The K562 and K562/ADR cells were infected by virus encapsulated with CSIV-TRE-RfA-CMV-KT-GnT-III plasmid. *A*, the expression levels of GnT-III were analyzed by immunoblot with an anti-GnT-III antibody, and α-tubulin served as a loading control. *B*, the same amounts of cell lysates were analyzed by immunoblot with the lectin E4-PHA, which preferentially recognizes the bisected *N*-glycans. *C*, effects of GnT-III on mRNA expression levels of three main subfamilies of ABC transporters, ABCB1 (P-gp), ABCC1, and ABCG2 were detected *via* RT-PCR. GAPDH was used as a control. *D*, the expression levels of P-gp were verified by immunoblot with anti-P-gp antibody; α-tubulin served as the control. ABC, ATP-binding cassette; ADR, adriamycin-resistant; GnT-III, N-acetylglucosaminyltransferase III; P-gp, P-glycoprotein.

To understand the impact that the downregulation of GnT-III exerts on the drug resistance of K562/ADR cells, we carried out a transfection approach to the overexpression of GnT-III in both K562 and K562/ADR cells *via* viral infection encapsulating the CSIV-TRE-RfA-CMV-KT-GnT-III plasmid and used FACSAria II to establish stable K562-GnT-III and K562/ADR-GnT-III cell lines after sorting. The expression levels of GnT-III were clearly increased in K562-GnT-III and K562/ADR-GnT-III cells, compared with control cells (Fig. 3*A*). The expression levels of endogenous GnT-III were very low, and only a faint band was detected in K562 cells, but none was detected in the K562/ADR cells. Lectin blotting also showed that the reactive abilities with E4-PHA were significantly enhanced in the K562-GnT-III and K562/ADR-GnT-III cells, which further indicated that GnT-III was successfully overexpressed in these two cells (Fig. 3*B*).

The resistance of cancer cells to anticancer drugs is usually due to the enhanced efflux of drugs by ABC transporters such as ABCB1 (P-gp) and others, as shown in Figure 3*C*. RT-PCR analysis has shown that the expression levels of P-gp, but not the other two subfamilies of the ABC transporters ABCC1 and ABCG2, were greatly enhanced in K562/ADR cells compared with that in K562 cells. It is noteworthy that the overexpression of GnT-III in K562/ADR cells substantially suppressed the expression levels of P-gp, which was also observed in the K562-GnT-III cells when compared with control cells (Fig. 3*C*). This tendency was also confirmed *via* Western blotting with anti-P-gp antibody (Fig. 3*D*). These results suggest that GnT-III negatively regulates P-gp expression.

### Overexpression of GnT-III reversed chemoresistance in K562/ADR cells

**Figure 4:**
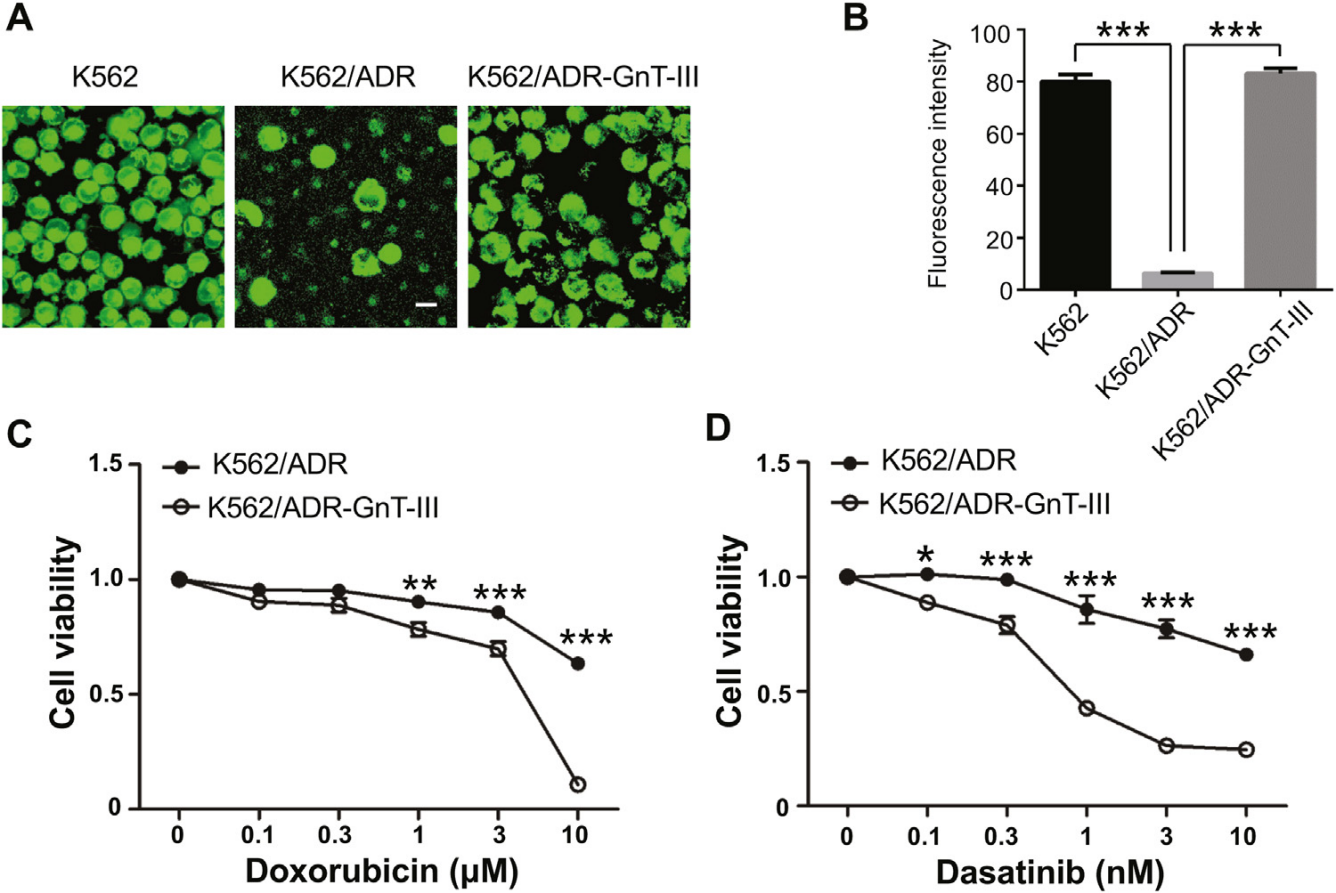
**GnT-III reversed chemoresistance by reducing the drug efflux.** Equal amounts of fluorescent dye were added to each cell line with the same cell numbers. After incubation for 1 h, the fluorescence intensities were assessed. *A*, a representative image of fluorochrome accumulation in cells was captured *via* confocal microscope. *B*, a fluorescence plate reader was used to quantitatively analyze the fluorescence intensities. The data are presented as the mean ± S.E.M (n = 3 in each group), one-way ANOVA; ∗∗∗*p* < 0.001. Scale bar: 10 μm. *C* and *D*, the K562/ADR and K562/ADR-GnT-III cells were cultured in the presence of doxorubicin and dasatinib at the indicated concentrations for 4 and 3 days, respectively, and then cell viabilities were measured *via* MTT assay. The inhibitory ratios for cell viabilities were normalized to that of each group without a drug (0 μM) and was set as 1. Data are reported as the mean ± S.E.M (n = 3), two-way ANOVA; ∗*p* < 0.05; ∗∗*p* < 0.01; ∗∗∗*p* < 0.001. ADR, adriamycin-resistant; GnT-III, N-acetylglucosaminyltransferase III.

To further investigate the effects of GnT-III on drug efflux, a drug retention experiment was performed. Equal amounts of fluorochromes were added to indicated cells followed by incubation for 1 h, and then the intracellular accumulation of fluorescence was visualized *via* confocal microscopy (Fig. 4*A*). Clearly, a higher amount of fluorescence remained in both the K562 and K562/ADR-GnT-III cells, compared with that in K562/ADR cells. Further, the fluorescence intensities were quantified by analysis using a fluorescence plate reader. This result showed that the fluorescence intensity in K562/ADR cells was significantly lower than that in K562 cells and that the overexpression of GnT-III rescued fluorescence accumulation (Fig. 4*B*). In addition, the cell viabilities were examined by exposure to DOX for 4 days (Fig. 4*C*) and to dasatinib (Fig. 4*D*) for 3 days at indicated concentrations. Overexpression of GnT-III significantly decreased the chemoresistance in K562/ADR-GnT-III cells, compared with that in K562/ADR cells. Taken together, these results suggest that GnT-III negatively regulates chemoresistance by reducing P-gp expression.

### The upregulation of P-gp expression induced by activating the NF-κB pathway was suppressed by the overexpression of GnT-III

**Figure 5:**
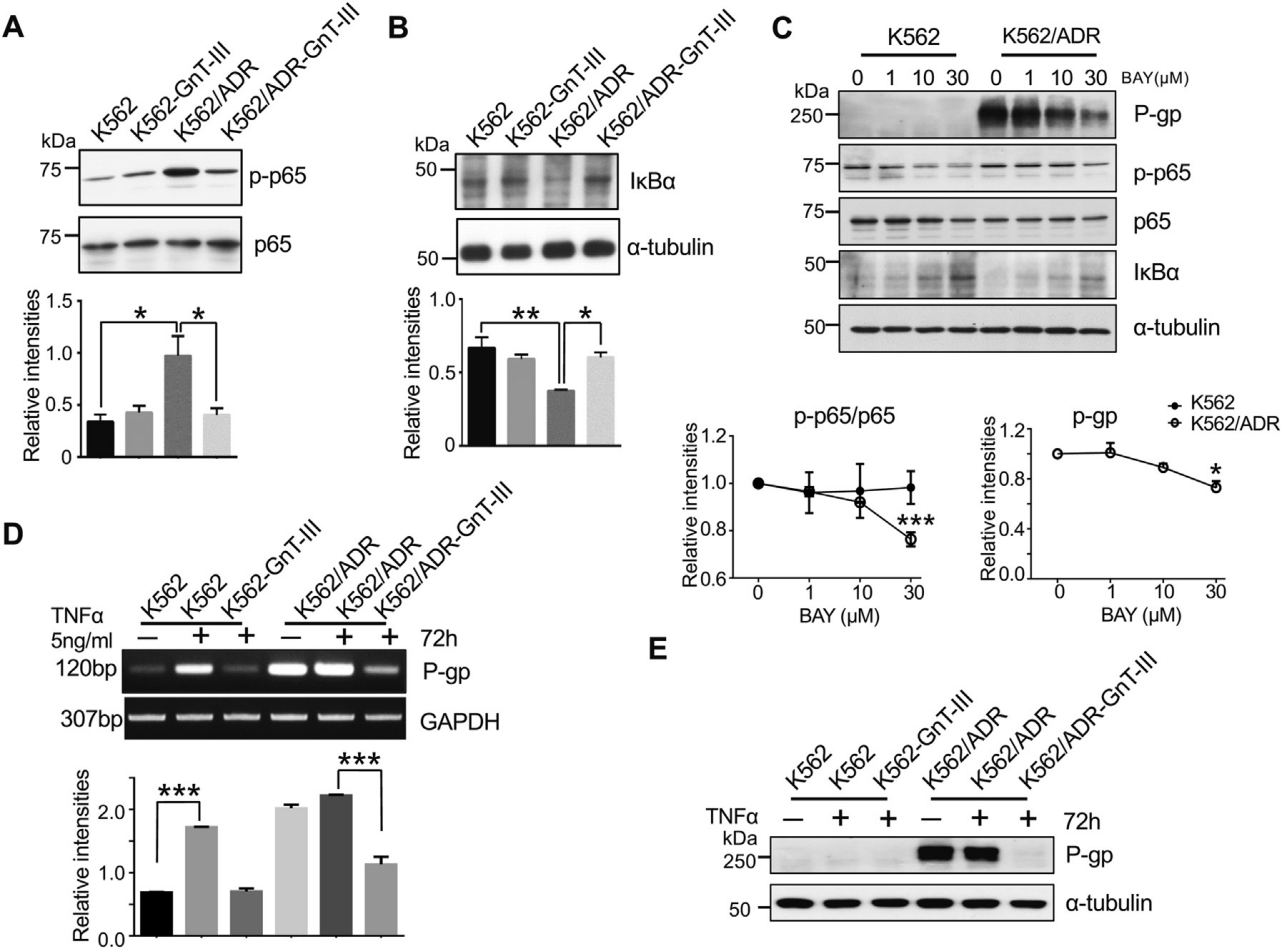
**Effects of GnT-III on TNFα/NF-κB signaling and P-gp expression.***A*, the expression levels of p65 and p-p65 in K562 and K562/ADR cells transfected with or without GnT-III were examined by immunoblotting with the indicated antibodies. *B*, the expression levels of IkBα in the indicated cells were examined by immunoblotting with anti-IkBα antibody, and α-tubulin was used as a loading control. Results are presented as the mean ± S.E.M of three independent experiments, one-way ANOVA; ∗*p* < 0.05, ∗∗*p* < 0.01. *C*, the K562 and K562/ADR cells were cultured with BAY 11-7082, an inhibitor of NF-κB signaling, for 24 h at the indicated concentrations. Equal amounts of cell lysates were detected by indicated antibodies, and α-tubulin served as a loading control. The relative intensities showed the ratios of either p-p65/p65 or P-gp/α-tubulin at each point against the ratio at 0 μM of BAY, which was set as 1. The data were presented as the mean ± S.E.M of three independent experiments, one-way ANOVA; ∗*p*< 0.05, ∗∗∗*p*< 0.001. *D*, the indicated cells were stimulated with TNFα (5 ng/ml) for 72 h. The mRNA expression levels of P-gp were analyzed *via* RT-PCR, and GAPDH was used as a loading control. The results are quantitatively presented as the mean ± S.E.M of three independent experiments, one-way ANOVA; ∗∗∗*p* < 0.001. *E*, the protein levels of P-gp were analyzed *via* Western blot and α-tubulin served as a loading control. ADR, adriamycin-resistant; GAPDH, glyceraldehyde-3-phosphate dehydrogenase; GnT-III, N-acetylglucosaminyltransferase III; P-gp, P-glycoprotein; TNFα, tumor necrosis factor α.

Next, we investigated the underlying molecular mechanisms by which GnT-III regulates P-gp expression and reverses MDR. Several studies have reported that the expression of P-gp could be regulated by the NF-κB pathway (12). Thus, to understand whether the NF-κB pathway was involved in the regulation of P-gp in cells, we first examined cellular signaling in the NF-κB pathway. This signaling was accomplished *via* phospho-p65 (p-p65), active signaling, and IkBα, which is an inhibitor protein for NF-κB. As shown in Figure 5*A*, the p-p65 levels were obviously increased in K562/ADR cells compared with that in K562 cells, which was attenuated after the overexpression of GnT-III in these cells. By contrast, the expression patterns of IkBα were opposite that of p-p65 (Fig. 5*B*). Then, we examined the effects of NF-κB signaling on P-gp expression in the present model. As expected, the expression levels of P-gp and p-p65/p65 ratios were significantly suppressed in the K562/ADR cells, while the levels of IκBα were increased when using the NF-κB inhibitor BAY 11-7082 to inhibit the ubiquitination of IκBα in a concentration-dependent manner (Fig. 5*C*).

It is well known that proinflammatory stimuli, such as TNFα can activate NF-κB signaling (42). Moreover, the TNFα/NF-κB signaling pathway mediates the expression of P-gp. Then, we investigated the effects that TNFα-mediated signaling exerts on the induction of P-gp. TNFα stimulation greatly upregulated P-gp expression, which was substantially suppressed by the overexpression of GnT-III analyzed *via* RT-PCR (Fig. 5*D*) and Western blot (Fig. 5*E*). It is unclear why the P-gp protein in the K562 cells remained at less than detectable levels, even during TNFα stimulation. We suspect the existence of different mechanisms that could be involved with P-gp stability and/or proteasomal degradation pathways or the existence of other related factors in K562 cells and K562/ADR cells. The exact mechanism will require further study in the future. However, these results still indicate that the expression of P-gp was upregulated after stimulation by TNFα and activation of the NF-κB pathway and that GnT-III negatively regulates MDR mainly *via* the TNFα/NF-κB pathway.

### GnT-III specifically modified TNFR2

**Figure 6:**
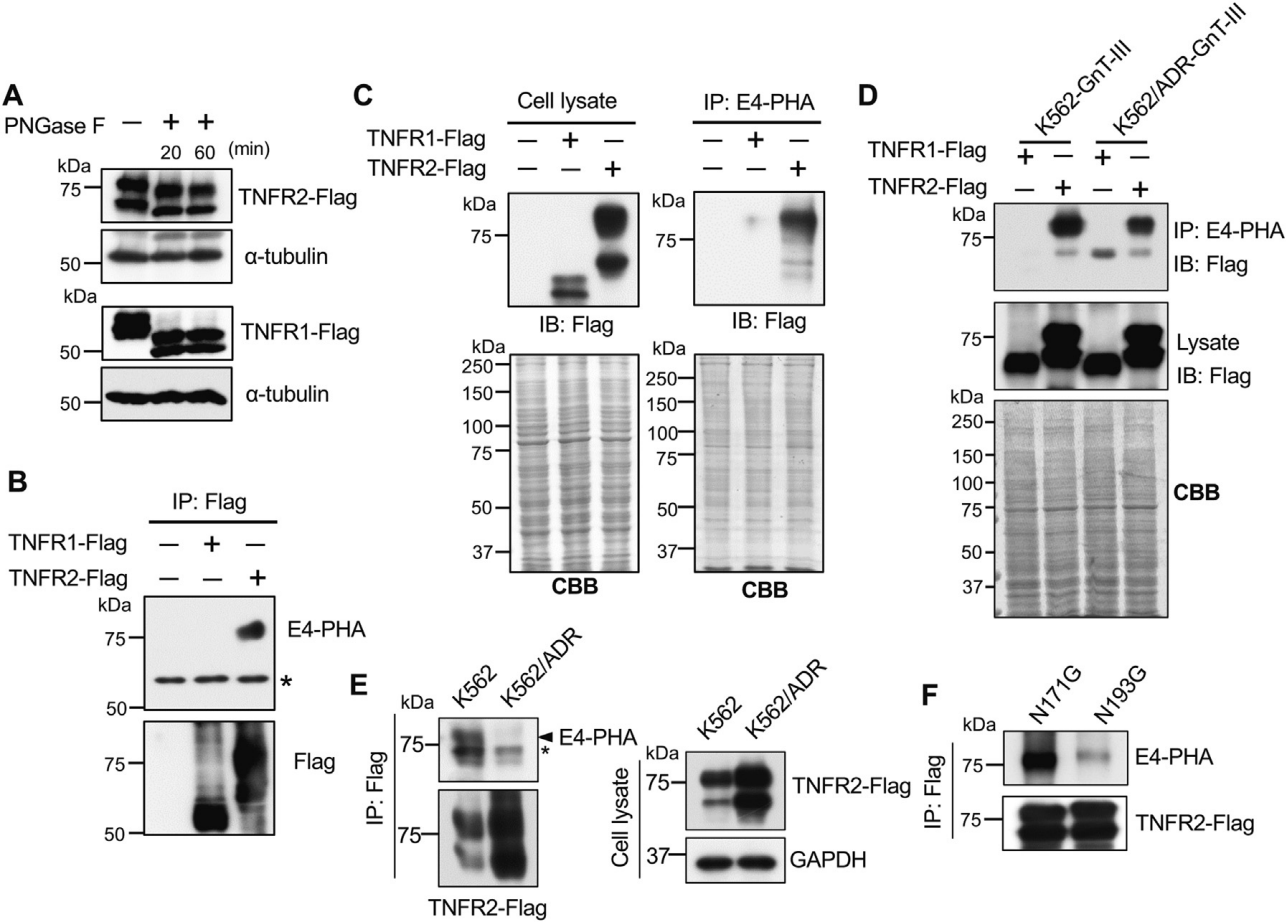
**GnT-III modified TNFR2, but not TNFR1.** The pcDNA3.1-RfA-TNFR1 and pcDNA3.1-RfA-TNFR2 plasmids containing a Flag tag were transiently transfected into 293T cells (*A*–*C* and *F*) or K562/ADR (*D* and *E*), and cell lysates were extracted after transfection for 48 h. *A*, equal amounts of cell lysates were treated with or without PNGase F for 20 min or 60 min and analyzed by immunoblotting with anti-Flag antibody. *B*, the cell extracts were immunoprecipitated (IP) with anti-Flag antibody subjected to 7.5% SDS-PAGE gel and immunoblotted with E4-PHA. The *asterisk* indicates nonspecific bands. *C* and *D*, cell lysates of 293T cells (*C*), K562-GnT-III and K562/ADR-GnT-III cells (*D*) were IP with E4-PHA agarose followed by immunoblotting with anti-Flag antibody. Coomassie brilliant blue staining showed the same amounts of proteins in each lane, which was used as a loading control. The experiments were independently repeated at least three times. *E* and *F*, the expression vectors containing wildtype TNFR2 or the N-glycosylation mutant of TNFR2 (Asn171G or Asn193G) tagged with Flag were transiently transfected into K562 or K562/ADR (*E*) or 293T cells (*F*), respectively. The same amounts of cell lysates extracted after transfection for 48 h, were immunoprecipitated with anti-Flag antibody, which was then followed by blotting with E4-PHA or anti-Flag antibody (upper panel). The *asterisk* indicates the nonspecific bands. Equal amounts of cell lysates were immunoblotted with anti-Flag antibody (*lower panel*). GAPDH was used as a loading control. ADR, adriamycin-resistant; CBB, Coomassie brilliant blue; GnT-III, N-acetylglucosaminyltransferase III; TNFR1, TNF receptor 1; TNFR2, TNF receptor 2.

TNFα exerts its signal transduction by stimulating receptors TNFR1 and TNFR2, which contain three and two potential *N*-glycosylation sites, respectively. At this point, in order to understand how the expression of GnT-III could regulate P-gp expression *via* the TNFα/NF-κB pathway, we initially investigated whether TNFR1 and TNFR2 could be modified by GnT-III. As described in the Methods section, we constructed human pcDNA3.1-RfA-TNFR1 and pcDNA3.1-RfA-TNFR2 plasmids containing a Flag tag. After transient transfection in 293T cells, cell lysates were digested with peptide *N*-glycosidase F (PNGase F), which removes all forms of *N*-glycan. The results of Western blotting with anti-Flag antibody showed that both TNFR1 and TNFR2 have two bands, which were shifted after treatment with PNGase F, which suggests that both were modified by *N*-glycans (Fig. 6*A*). Surprisingly, the results of lectin blotting with E4-PHA, which preferentially recognizes bisected *N*-glycans, showed that TNFR2, but not TNFR1, was modified by GnT-III (Fig. 6*B*). To further confirm this observation, cell lysates were immunoprecipitated with E4-PHA-agarose, and then, the immunoprecipitates were western blotted with anti-Flag antibody. It was possible to consistently detect only TNFR2 (Fig. 6*C*). Furthermore, to confirm whether the specific modification also occurred in K562 cells, both K562 and K562/ADR cells were transiently transfected with pcDNA3.1-RfA-TNFR1 and pcDNA3.1-RfA-TNFR2 plasmids. And then, cell lysates were immunoprecipitated with E4-PHA-agarose and blotted with anti-Flag antibody. Consistently, TNFR2, not TNRF1, could be detected (Fig. 6*D*). Moreover, compared with K562 cells, the lectin blotting with E4-PHA showed that the modification by bisected *N*-glycans on TNFR2 was significantly suppressed in K562/ADR cells (Fig. 6*E*). There are two potential N-glycosylation sites (Asn171 and Asn193) on human TNFR2. In order to investigate the specific modification of GnT-III, we constructed N-glycosylation mutants of TNFR2 (Asn171 G and Asn193 G) and found that GnT-III preferentially modifies the Asn193 site rather than the Asn171 site. These data strongly suggest that the deficiency of bisected *N*-glycans on TNFR2 may be involved in the acquisition of chemoresistance.

### The lack of bisected N-glycans induced TNFR2 trimerization

**Figure 7:**
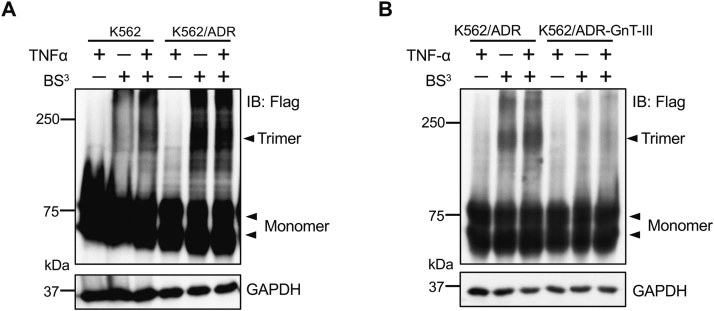
**Lack of bisected *N*-glycans on TNFR2 induced its autotrimerization, which was blocked by GnT-III expression.***A*, the K562 and K562/ADR cells were infected by a virus encapsulated with CSIV-TRE-RfA-CMV-KT-TNFR2 plasmid containing a Flag tag, which then was treated with or without TNFα (5 ng/ml), and was further incubated with or without BS^3^, which is a chemical cross-linker that is described in the Experimental procedures section. Equal amounts of cell lysates were subjected to 7.5% SDS-PAGE and blotted with anti-Flag antibody to detect TNFR2 monomer and trimer. *B*, the K562/ADR cells were cotransfected with pLX303-GnT-III and TNFR2. The same amounts of cell lysates were immunoblotted with anti-Flag antibody. GAPDH was used as a loading control. ADR, adriamycin-resistant; GnT-III, N-acetylglucosaminyltransferase III; TNFα, tumor necrosis factor α; TNFR1, TNF receptor 1; TNFR2, TNF receptor 2.

The initial step for signal transduction mediated by members of the TNFR superfamily is ligand-induced trimerization of the receptors (43). Therefore, we attempted to identify trimer complexes using a BS^3^ linker with or without TNFα stimuli. We used a viral infection to overexpress TNFR2 *via* encapsulation of the CSIV-TRE-RfA-CMV-KT-TNFR2 plasmid with a Flag tag in K562 and K562/ADR cell lines, which were treated with or without BS^3^ for the cross-linking of cell-surface proteins. As shown in Figure 7*A*, the trimerization formation of TNFR2 could be induced *via* TNFα treatment of K562 cells since the molecular size of 210 kDa was approximately three times the monomer size, which could also suggest that it is the upper band of TNFR2 (at approximately 75 kDa, which is the so-called mature form) rather than the lower band (around 55 kDa, the so-called immature form) that participates in trimerization formation. By contrast, trimerization was clearly observed in the K562/ADR cells even without TNFα stimulation, which was greatly attenuated in the K562/ADR-GnT-III cells (Fig. 7*B*). Taken together, these results suggest that the expression of GnT-III negatively regulates the trimerization of TNFR2 on the cell surface.

### Deficiency of the TNFR2 gene downregulated P-gp expression, but it upregulated GnT-III expression

**Figure 8:**
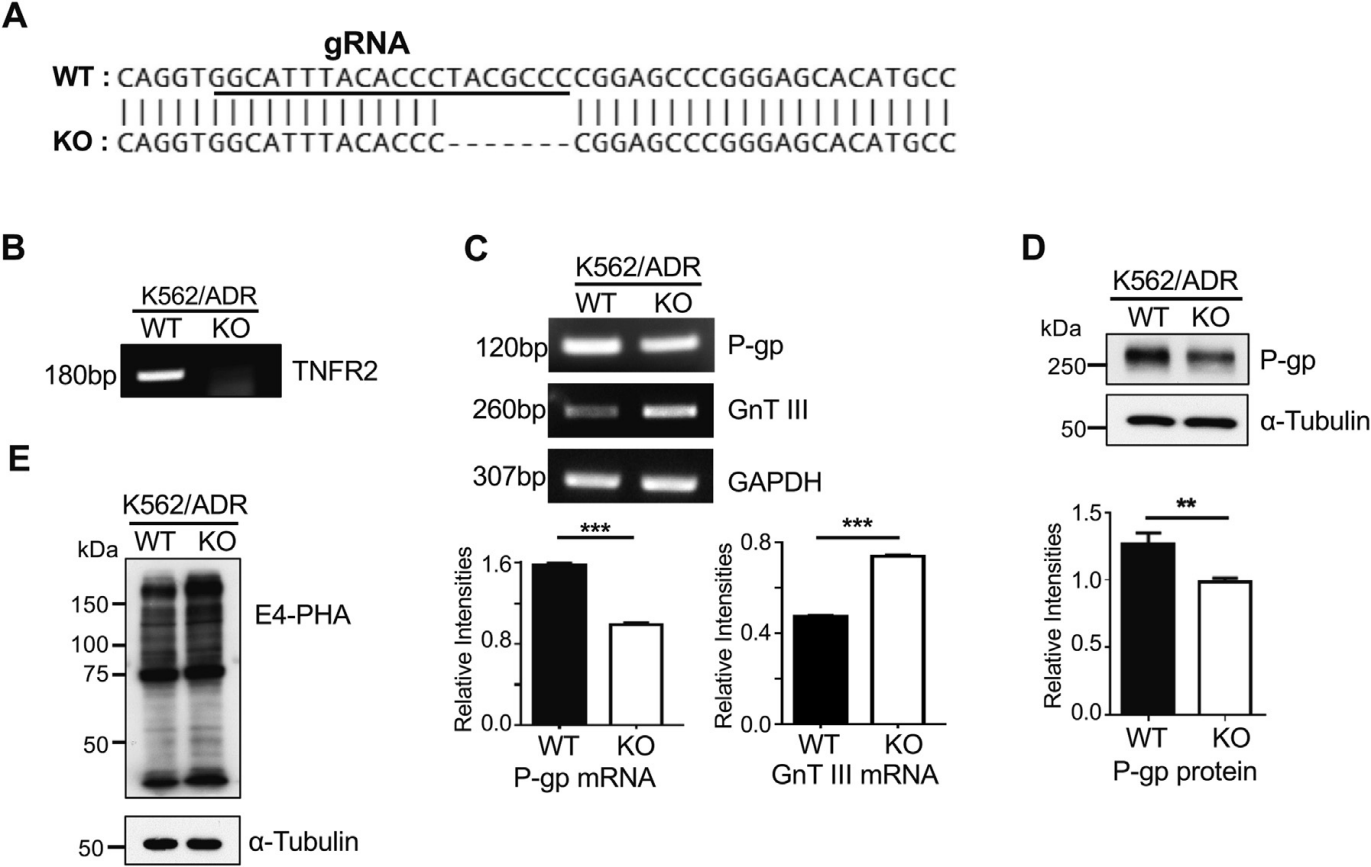
**Effects of TNFR2-KO on P-gp and GnT-III expression.***A* and *B*, the TNFR2-KO cells were established by guide RNA sequence (*underline*) and confirmed *via* their genomic sequences (*A*) and by RT-PCR (*B*) described in the Experimental procedures section. *C*, the mRNA levels of P-gp and GnT-III were detected *via* RT-PCR, and GAPDH was used as an internal control. The data are reported as the mean ± S.E.M (n = 3) from three independent experiments, one-way ANOVA; ∗∗∗*p* < 0.001. *D*, the expression levels of P-gp were verified by immunoblot with anti-P-gp antibody, and α-tubulin served as the control. The data are reported as the mean ± S.E.M (n = 3) from three independent experiments, one-way ANOVA; ∗∗*p* < 0.01. *E*, equal amounts of cell lysates of WT and the TNFR2-KO K562/ADR cells were stained with E4-PHA, and α-tubulin was used as a loading control. ADR, adriamycin-resistant; GnT-III, N-acetylglucosaminyltransferase III; P-gp, P-glycoprotein; TNFR2, TNF receptor 2.

To further understand the relationships among TNFR2, GnT-III, and P-gp, we established a TNFR2-KO K562/ADR cell line. The results obtained from DNA sequencing showed that seven bases were deleted (Fig. 8*A*), which was further confirmed *via* RT-PCR (Fig. 8*B*). Compared with the K562/ADR cells, the mRNA level of P-gp was downregulated in the TNFR2-KO cells (Fig. 8*C*), which was also verified by Western blotting with anti-P-gp antibody (Fig. 8*D*). However, the mRNA level of GnT-III was obviously upregulated in the TNFR2-KO cells (Fig. 8*C*). Also, the expression levels of the bisected N-glycans detected by E4-PHA lectin were obviously increased in the TNFR2-KO cells, compared with levels in the K562/ADR cells (Fig. 8*E*). These data further suggest the importance of TNFR2-mediated signaling for the regulation of P-gp expression and also for GnT-III expression.

## Discussion

**Figure 9:**
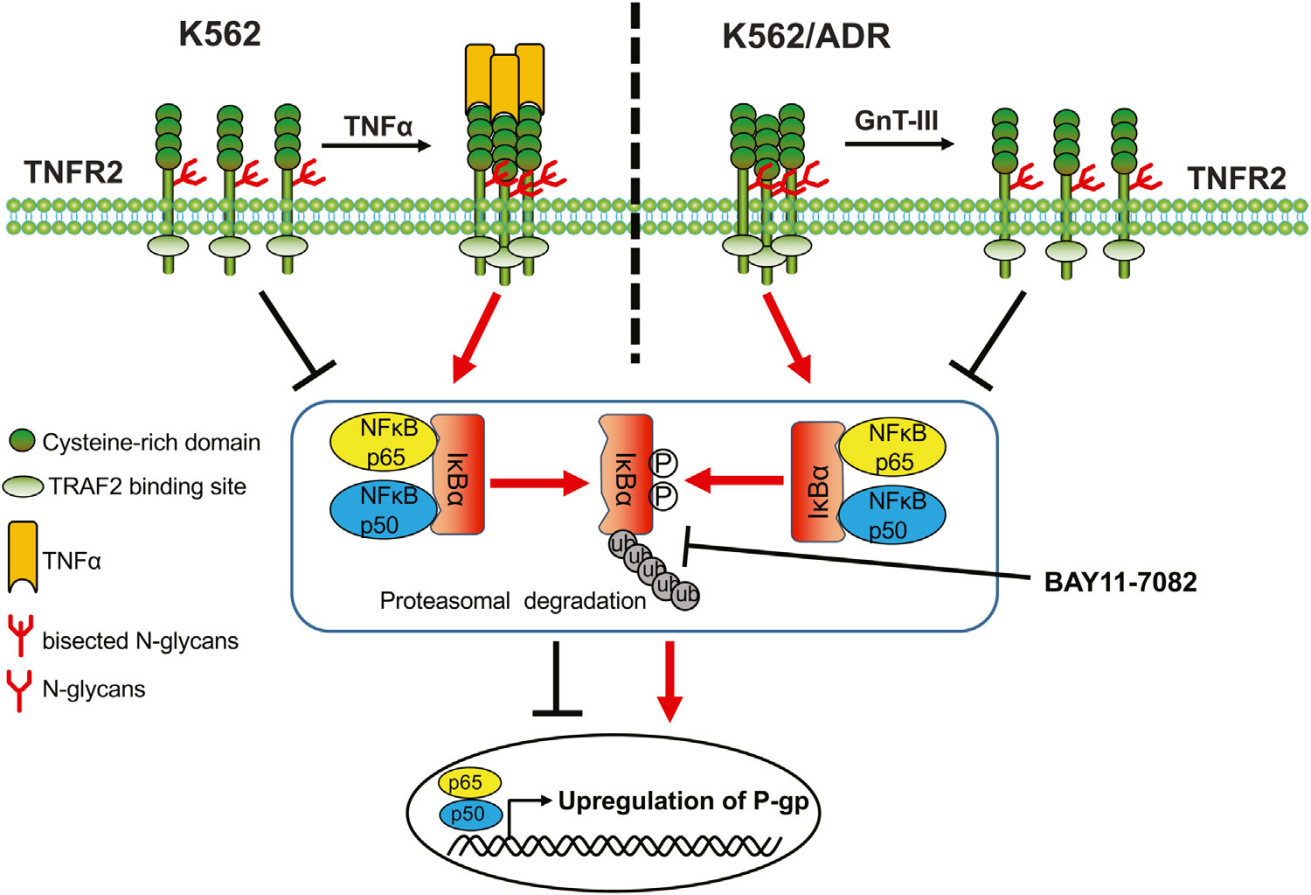
**Schematic diagram of the proposed molecular mechanism for negative regulation of MDR *via* GnT-III.** In the present study, we found that GnT-III expression negatively regulates P-gp expression *via* a TNFR2-mediated NF-κB signal pathway. The K562 cells highly expressed GnT-III, and TNFα is required for the trimeric formation of TNFR2 on the cell surface, which then subsequently induces NF-κB signaling for the upregulation of P-gp. By contrast, once the K562 cells get chemoresistance such as the K562/ADR cells, the expression levels of GnT-III are substantially suppressed. The deficiency of bisected *N*-glycans on TNFR2 strongly induces its autotrimerization and then activates NF-κB signaling for the upregulation of P-gp. Furthermore, the restoration of GnT-III in the K562/ADR cells impedes TNFR2 autotrimerization and suppresses NF-κB signaling for the regulation of P-gp expression, which is similar with the effects of BAY 11-7082 as an NF-κB inhibitor to inhibit ubiquitination of IkBα. The present study could provide valuable direction for the development of a new therapy to reverse MDR and resensitize tumor cells to antineoplastic agents. The *red arrows* show the activation of NF-κB signaling, whiles *black lines* show the inhibition of NF-κB signaling. ADR, adriamycin-resistant; GnT-III, N-acetylglucosaminyltransferase III; P-gp, P-glycoprotein; TNFα, tumor necrosis factor α; TNFR2, TNF receptor 2.

MDR is a fundamental problem that limits the effectiveness of many chemotherapies currently used for cancer treatment. In the present study, we clearly showed that the expression of GnT-III plays an important role in chemoresistance by regulating P-gp expression *via* the TNFα/NF-κB signaling pathway. We used a human CML cell line, K562/ADR cells, as the MDR cell model, and found the following four results: (1) expression levels of GnT-III and its product that bisects GlcNAc were suppressed in the K562/ADR cells; (2) the suppression of GnT-III induced autotrimerization of TNFR2 and NF-κB signaling for the upregulation of P-gp expression; (3) the overexpression of GnT-III in K562/ADR cells rescued these phenomena, as observed in the parental K562 cells; and (4) the deficiency of TNFR2 decreased P-gp expression, but it increased GnT-III expression (Fig. 9). It is noteworthy that the decreased expression levels of bisected *N*-glycans catalyzed by GnT-III were not restricted to K562/ADR cells, which has also been observed in several other drug-resistant cell lines (31, 38, 39). Consistently, the expression of bisecting GlcNAc in glioma cells increased the P-gp–mediated cell response to vinblastine (44). Thus, the present study outlines the novel underlying mechanism responsible for the inhibition of chemoresistance by GnT-III and provides new insights into the role of *N*-glycosylation in the regulation of cancer MDR.

A prevalent mechanism of cancer MDR is the upregulation of P-gp, which is responsible for effluxing toxins and xenobiotics from cells. Therefore, P-gp has long been recognized as a viable target to overcome MDR in cancer treatment. The results of the present study consistently showed that the expression of P-gp in drug-resistant K562/ADR cells was significantly increased compared with that in the parent K562 cells (Fig. 1*A*). Several P-gp inhibitors or reversal agents have been applied in clinical trials for cancer treatment. For example, ivermectin, a macrolide antiparasitic agent, is known to reverse the resistance of tumor cells to chemotherapeutics by reducing the expression of P-gp (45). Moreover, we found that an overexpression of GnT-III in K562/ADR cells significantly suppressed P-gp expression (Fig. 3) and reversed chemoresistance to DOX and dasatinib (Fig. 4). We further investigated the potential mechanisms of how GnT-III regulates P-gp expression. NF-κB is one of the major transcription factors for the human P-gp gene (18). The induction of the NF-κB pathway has been associated with an increase in P-gp gene expression, while the inhibition of NF-κB has inversely led to a downregulation of P-gp expression (12, 46, 47). Moreover, NF-κB directly binds to the P-gp gene promoter to upregulate P-gp expression at the transcriptional level to induce drug resistance (11, 12). Based on these observations, we speculated that GnT-III could regulate P-gp expression *via* the NF-κB pathway. As expected, the restoration of GnT-III in K562/ADR cells led to an inhibition of the NF-κB pathway (Fig. 5), which was consistent with the suppression of P-gp expression caused by the overexpression of GnT-III in K562/ADR cells (Fig. 3). In addition, blockage of the NF-κB signaling pathway by BAY 11-7082, an NF-κB inhibitor, downregulated P-gp expression (Fig. 5*C*). Therefore, we concluded that GnT-III regulates P-gp expression *via* NF-κB signaling.

The next issue concerned the question of how GnT-III affects NF-κB signaling because GnT-III only modifies extracellular domain of glycoproteins. NF-κB is a target of TNFα-mediated signaling (48). Furthermore, the present study and several others have reported a strong relationship between TNFα and P-gp (13, 14, 15, 16, 17, 18) (Fig. 5*D*). TNFα is activated by binding to two different types of receptors: TNFR1 and TNFR2. Previous studies have demonstrated the importance of *N*-glycosylation on TNFR1 (20, 21), the loss of which suppresses its binding to TNFα and represses TNFα-mediated NF-κB signaling (20). Thus, we speculated that TNFR1 could be modified by GnT-III. However, it is interesting that our results show that rather than TNFR1, it is TNFR2 that is modified by bisected *N*-glycans. Consistently, the expression of TNFR2 containing the bisected *N*-glycans was clearly detected in the K562 cells, but this expression was undetectable in K562/ADR cells (Fig. 6, *D* and *E*). Reports have associated TNFR2 with cancer chemoresistance, and the blockage of TNFR2 in resistant cells has restored sensitivity to drug treatment (49). TNF family receptors are known to exist as preassembled oligomers on the cell surface in the absence of ligands and are activated by ligands to form a trimer (50). It is noteworthy that the loss of modification in the bisecting of GlcNAc on TNFR2 strongly induced autotrimerization and downstream signaling in K562/ADR cells, and this phenomenon was suppressed by the overexpression of GnT-III (Fig. 7). Furthermore, a deficiency of the TNFR2 gene significantly suppressed P-gp expression but enhanced GnT-III expression (Fig. 8). These findings may help us to understand how GnT-III regulates the NF-κB signaling and then affects the expression of P-gp. Of course, we could not exclude the influences of GnT-III expression from other receptors. In fact, Yoshimura *et al.* (51) reported that the modification of bisected *N*-glycans protected K562 cells from attacks by natural killer cells, wherein the receptor is clearly different from that of TNFR2.

We were interested to understand why GnT-III specifically modifies the two potential *N*-glycosylation sites of TNFR2 and preferentially modifies N193 rather than N171 (Fig. 6*F*) but does not modify the three potential *N*-glycosylation sites of TNFR1. The selective modification by GnT-III was also observed in some specific sites of glycoproteins such as integrin α5, wherein GnT-III specifically modified the site-4 from among 14 other potential *N*-glycosylation sites (52). In addition, the phenomenon was also observed in the ɑ2,3-sialylation of *N*-glycans catalyzed by three α2,3-sialyltransferases, ST3GAL3, ST3GAL4, and ST3GAL6, which differ in their modification of target proteins and in their regulation of cell biological functions (53). There currently is no detailed information available regarding those observations, but several explanations have been proposed. First, *N*-glycosylation occurs on TNFR2 because it provides the easiest access for GnT-III. Due to the current lack of information on the TNFR2 crystal structure, however, this hypothesis cannot be proven. Second, GnT-III may associate with other molecules, which could define the specificities for protein or peptide substrates. Indeed, caveolin-1 is known to co-localize with GnT-III to regulate its localization and activity (54). Very recently, Osuka *et al.* (55) reported that the N domain of GnT-V plays a critical role in the recognition of glycoprotein substrates, which suggests a glycosyltransferase may selectively modify target glycoproteins. Third, glycosyltransferases selectively modify target glycoproteins such as TNFR2 and TNFR1 either through different pathways or *via* functionally distinct Golgi units referred to as the “zones” of mitochondria (56) or of the Golgi apparatus (57, 58, 59). These results, taken together, suggest that glycosyltransferase complex formation may play a crucial role in determining both activity and substrate specificity. Details of the molecular mechanism, however, will require further study.

Thus far, the reason for the downregulation of GnT-III in MDR remains unclear. Intriguingly, cell density on a culture dish affected GnT-III expression, and higher cell densities were found to induce GnT-III mRNA (60). In addition, the E-cadherin–α-catenin complex was shown to be involved in the process (61). Meanwhile, Wnt/β-catenin signaling negatively regulated both GnT-III expression and the bisecting of GlcNAc *via* the product of this expression (62). It could be a coincidence that Wnt/β-catenin signaling is related to drug resistance. The activation of Wnt/β-catenin signaling increases P-gp expression, which results in chemoresistance (63). The relationship between NF-κB and Wnt/β-catenin signaling remains elusive and will require further study. In addition, epigenetic regulation could also be important for GnT-III expression. DNA hypomethylation of the GnT-III promoter is involved in its upregulation in ovarian cancer cells (64), as well as in other types of cancers (65). The details of the molecular mechanism for the downregulation of GnT-III in MDR will require further study.

As far as we could ascertain, the present study is the first to clearly demonstrate that GnT-III expression negatively regulates P-gp expression *via* the TNFR2/NF-κB signaling pathway, which contributes to cancer chemoresistance. This finding may provide a hint for the development of a new therapy to reverse MDR and resensitize tumor cells to antineoplastic agents.

## Experimental procedures

### Antibodies and reagents

The experiments were performed using the following antibodies: mouse mAb against IκBα (sc-1643), NF-κB p65(sc-8008), and rabbit mAbs against glyceraldehyde-3-phosphate dehydrogenase (GAPDH) (sc-25778), which were obtained from Santa Cruz Biotechnology; rabbit mAb against Phospho-NF-κB p65 (Ser536) (3033) and a peroxidase-conjugated secondary antibody against rabbit (7074S), which are from Cell Signaling Technology; mouse mAbs against α-tubulin (T6199) and peroxidase-conjugated secondary agents against mouse (AP124P), which were acquired from Sigma-Aldrich; rabbit mAb against GnT-III (33A8), which was obtained from FUJIREBIO Inc; mouse mAbs against FLAG (F1804) and anti-FLAG conjugated to agarose (A2220), which is from Sigma-Aldrich; mouse mAb against ABCB1(P-gp) (clone C219), which is from Biolegend; biotinylated *Phaseolus vulgaris* erythroagglutinin (E4-PHA; B-1385), biotinylated *Phaseolus Vulgaris Leucoagglutinin* (L4-PHA; B-1115–2), *Concanavalin* A (ConA; B-1005), *Sambucus nigra* agglutinin (SNA; B-1305), *Datura stramonium* agglutinin (DSA; B-1185), and an ABC kit, which were acquired from Vector Laboratories; E4-PHA agarose (J311), which is from J-OIL MILLS; Recombinant human TNFα (H8916), BAY 11-7082 (B5556), dasatinib (SML2589), and doxycycline hyclate (D9891), which were acquired from Sigma-Aldrich; DOX (adriamycin) (S1208), which was purchased from Selleck Chemicals; BS^3^ linker (A39266), which was obtained from Thermo Scientific; and a multiple drug resistance microtiterplate assay kit (M1580), which is from Marker Gene Technologies.

### Cell cultures

The leukemia cell line K562 was obtained from the Cell Resource Center for Biomedical Research, Tohoku University. Cells were grown at 37 °C in a CO_2_ incubator in RPMI 1640 medium containing 10% fetal bovine serum (endotoxin level 10 EU/ml/ml) and 1% antibiotic/antimycotic agent (Gibco).

The K562/ADR cells were obtained from adriamycin (DOX)-resistant K562 cells. The parental K562 cells were treated with DOX concentrations in a stepwise increase (0.5 nM for each step) of from 5 to 10 nM during a 4-month incubation. The cells that survived served as the K562/ADR cells. Cell viability was measured by counting, and the cells stained with 0.2% trypan blue were excluded.

### Establishment of TNFR2-KO cells

The pSpCas9(BB)-2A-GFP (PX458) plasmid was purchased from Addgene (PX458: Addgene #48138). The TNFR2-KO cells were established by guide RNA (5′- GGCATTTACACCCTACGCCC -3′) that targeted the human TNFR2 gene localized adjacent to Cas 9 in the pSpCas9(BB)-2A-GFP vector, and then the GFP-expressing cells were sorted out. Single cells were cultured for 3 weeks and were then confirmed *via* their genomic sequences. Genomic DNA was amplified *via* PCR using a forward primer (5′- CCCCAGCCATCATCAGTGC -3′) and a reverse primer (5′- AGGGCTATGCTCTTGCGCC -3′). And the TNFR2-KO cells were further confirmed by RT-PCR using a forward primer (5′- CATTTACACCCTACGCCCC -3′) and a reverse primer (5′- AGGGCTATGCTCTTGCGCC -3′).

### Construction of expression vector

We used the Gateway cloning system from Invitrogen for all overexpression experiments. The Gateway entry vectors were constructed as described previously (66). In brief, the cDNA of TNFR1 and TNFR2 were cloned from K562 cells and then inserted into pENTR 1A tagged with a 3× FLAG at the C terminus using the in-fusion method (Takara Bio Inc). The vectors of TNFR2 with altered N-glycosylation sites, which were indicated as N171G and N193G mutations, were constructed according to the in-fusion kit using the following primers: 5′- GTTCTCCGGCACGACTTCATCCACGGATATTTGC -3′, 5′- GTCGTGCCGGAGAACGTCCCCGGGGC -3′ and 5′- CCCTGGGGGCGCAAGCATGGATGCAGTCTGC -3′, 5′- CTTGCGCCCCCAGGGATGGCCACCACG -3′, respectively. The resultant cDNAs were sequenced to confirm the presence of the desired mutations.

The GnT-III pENTR/D-TOPO vector that was used was previously established in our lab (67). The resultant cDNAs in entry vectors were confirmed *via* DNA sequencing. Then, we used the Gateway cloning system kit (Invitrogen) to acquire all of the expression vectors. Briefly, using LR clonase (Invitrogen), the subcloned cDNAs in entry vectors were transferred into pcDNA3.1-RfA for a transient expression, CSIV-TRE-RfA-CMV-KT for a doxycycline hyclate–inducible overexpression and pLX303 for a stable expression. For transient transfection, generally, the expression vectors/PEI MAX (1 mg/ml in 0.2 M hydrochloric acid) were combined at a 1:3 ratio to the target cells. After incubation for 6 h, the conditioned medium was replaced with a normal culture medium and then further incubated for 48 h.

### Virus production and infection

Steps to accomplish virus production and infection were performed as described previously (53, 68). Briefly, the lentivirus vectors (CSIV-TRE-RfA-CMV-KT-TNFR1, CSIV-TRE-RfA-CMV-KT-TNFR2, CSIV-TRE-RfA-CMV-KT-GnT-III or pLX303-GnT-III) were co-transfected with psPAX2, pMD2G, and pCMV-tax into 293 T cells *via* PEI MAX. After transfection for 48 h, the lentivirus supernatants were filtered and then incubated with target cells in the presence of 10 μg/ml polybrene (Millipore Sigma). After infection for 48 h, the Kusabira Orangepositive cells (CSIV-TRE-RfA-CMV-KT) were sorted twice using FACSAria II (BD Bioscience), and the stable cell lines were used in subsequent studies. To select cells with a stable integration of pLX303-GnT-III in K562/ADR, the cells were treated with 10 μg/ml blasticidin for 5 days, and the cells that reacted with E4-PHA were selected *via* FACSAria II. The selected cells were then referred to as K562/ADR GnT-III cells.

### RT-PCR for mRNA expression analysis

The RNA was extracted using TRIzol Reagent (Invitrogen) and then reverse-transcribed using a PrimeScript RT reagent kit (Takara Bio Inc). The specific primer sequences are listed in Table 1. GAPDH was performed as a control. The reaction products were electrophoresed on 1.5% agarose gel in TBE buffer at 135 V for 30 min. Finally, the DNA ladder was visualized with ethidium-bromide staining under UV light.

**Table 1:** Primer sequences for RT-PCR

Target gene	Primer sequences (5′-3′)	
	Forward sequences	Reverse sequences
ABCB1	GCTGTCAAGGAAGCCAATGCCT	TGCAATGGCGATCCTCTGCTTC
ABCC1	AGGACACGTCGGAACAAGTC	GGAAGTAGGGCCCAAAGGTC
ABCG2	GTTCTCAGCAGCTCTTCGGCTT	TCCTCCAGACACACCACGGATA
GnT-I	TGACCAGCACCTCAAGTTTATC	CGGAACTGGAAGGTGACAATAC
GnT-II	AGAGTGCCCTGAATGTGATG	CACAGTCTCCAGCATGAAAGA
GnT-III	GCCGCGTCATCAACGCCATCAA	CAGGTAGTCGTCGGCGATCCA
GnT-IVa	GGCTATCACACCGATAGCTGGAG	TCCACCATTCCTTCTGCAACACC
GnT-IVb	ACAACCCTCAGTCAGACAAGGAGG	GGTACCCTCAGAAGCCCGCAGCTT
GnT-V	GACCTGCAGTTCCTTCTTCG	CCATGGCAGAAGTCCTGTTT
GAPDH	CGGAGTCAACGGATTTGGTCGTA	AGCCTTCTCCATGGTGGTGAAGAC

### Western blot, lectin blot, and immunoprecipitation

Total samples of protein were collected from cells using lysate buffer (20 mM Tris-HCl, pH 7.4, 150 mM NaCl, 1% Triton X-100) with protease and phosphatase inhibitors (Nacalai Tesque) and were quantified using a BCA protein assay kit (Pierce). Appropriate cell lysates were subjected to SDS-PAGE and transferred to a PVDF membrane (Millipore Sigma). The membranes were then incubated with indicated primary and secondary antibodies or with biotinylated lectins as indicated. Protein bands were visualized using the Immobilon Western Chemiluminescent HRP Substrate (Millipore Sigma).

For immunoprecipitation, appropriate cell lysates were incubated with 10 μl anti-Flag conjugated agarose or 20 μl anti-E4-PHA agarose and rotated for 2 h at 4 °C. Then, the immunoprecipitates were washed twice with lysis buffer, separated *via* SDS-PAGE, and subjected to Western blot analysis.

### Flow cytometry analysis

Cells (2 × 10^5^/well) were collected and washed with cold phosphate-buffered saline (PBS) on ice. Cells were incubated with or without biotinylated lectins (E4-PHA) in 0.1% BSA/PBS for 30 min and then stained with Alexa Fluor 647 for 25 min on ice. Then, cells were washed three times and resuspended in 1 ml of 0.1% BSA/PBS and analyzed using Attune flow cytometer (BD Biosciences).

### Cytotoxicity assay

Cells (K562/ADR and K562/ADR GnT-III; 8 × 10^3^/well) were seeded in 96-well plates and exposed to indicated concentrations of DOX for 4 days or to dasatinib for 3 days. Cell viability was detected by adding 10 μl of MTT solution (5 mg/ml) (Dojindo) to each well with subsequent incubation at 37 °C for 4 h. The plate was then centrifuged at 2000 rpm for 20 min, and the supernatant was removed. Subsequently, 100 μl of DMSO (Millipore Sigma) was added into each well at 37 °C for 5 min. Finally, cell viability was evaluated at an absorbance of 490 nm using a microplate reader (Infinite M1000, TECAN).

### Glycan analysis of cell membrane proteins *via* LC-ESI MS

*N*-Glycans from cell membrane proteins were released (69) and then labeled with TMT6 (Thermo Fisher Scientific) and finally analyzed *via* LC-ESI MS, according to previous procedures (70, 71) with some modifications as follows. Briefly, K562 and K562/ADR cells (1 × 10^7^ cells each) were centrifuged after homogenization using a polytron to remove nuclei and unbroken cells. The supernatant was ultracentrifuged, and then, the membrane pellet was mixed with ice-cold acetone after purification by phase partitioning. After centrifugation, the precipitated membrane proteins were dissolved and spotted onto PVDF membrane. After staining, the protein spots were excised from the PVDF membrane and placed into one of the wells in a 96-well plate. PNGase F (Roche) was added to the spots after blocking of PVDF membrane. The released *N*-glycans were reacted with TMT6. *N*-Glycans labeled with TMT6 were separated on a carbon column (5 μm HyperCarb, 1 mm I.D. × 100 mm, Thermo Fisher Scientific), and the eluate was introduced continuously into an ESI source (LTQ Orbitrap XL, Thermo Fisher Scientific). MS spectra were obtained in the positive ion mode using Orbitrap. For MS/MS analysis, the top three precursor ions were fragmented by HCD using stepped-collision energy *via* an Orbitrap. Monoisotopic masses were assigned possible monosaccharide compositions using a GlycoMod software tool (mass tolerance for precursor ions as [M + H]^+^ is ± 0.03 Da, https://web.expasy.org/glycomod/), and the proposed glycan structures were further verified through annotation using a fragmentation mass-matching approach based on the MS/MS data. Xcalibur software ver. 2.2. (Thermo Fisher Scientific) was used to show the base peak chromatogram and the extracted ion chromatogram and to analyze the MS and MS/MS data. The relative abundances (%) of each glycan structure were calculated by setting the total peak intensities of all detected *N*-glycans labeled with TMT6 in each extracted ion chromatogram as 100%.

### Cross-linking experiment

A cross-linking assay was performed as described previously (72). Briefly, cells were centrifuged and resuspended in culture medium incubated with or without human recombinant TNFα (5 ng/ml) for 5 min at 37 °C. Then, the cells were washed three times with PBS and treated with 1 mM BS^3^ for 30 min at room temperature. The reaction was stopped by adding 150 mM glycine-HCl (pH = 7.5) for 5 min on ice. The cells were lysed with RIPA buffer [Tris-HCl (pH = 7.5), 150 mM NaCl, 1% Triton X-100, 0.1% SDS]. Equal amounts of the lysates were subjected to electrophoresis under nonreducing (without β-mercaptoethanol) conditions, which was followed by analysis *via* western blotting with anti-Flag antibody.

### Intracellular accumulation of fluorochromes

To assay the drug efflux, cells were counted and suspended in culture media at a concentration of 1 × 10^6^ cells/ml/tube. And then, 1 μl of 8 μM fluorescent dyes (multiple drug resistance microtiterplate assay kit; Marker Gene Technologies, Inc) was added and incubated for 1 h at 37 °C. Cells were pelleted through centrifugation, washed three times with PBS, and then resuspended as cell pellets in 200 μl PBS. The intracellular accumulation of fluorochromes was analyzed using a microplate reader (Ex = 504 nm; Em = 538 nm, Thermo Fisher Scientific). To visualize the intracellular retention of fluorochromes, cells were transferred onto coverslips, and images were taken using a LSM900 confocal microscope (ZEISS ZEN 3.0).

### Statistical analysis

Results are presented as the mean ± S.E.M. Statistical significance was calculated *via* analysis of variance (ANOVA) test using GraphPad Prism 5.0 software (GraphPad Software, Inc). (∗, ∗∗, and ∗∗∗ represent *p*-values of <0.05, 0.01, and 0.001, respectively).

## Data availability

Glycomic raw data for glycan-structure analysis by LC-ESI MS have been deposited to the GlycoPOST; announced ID: GPST000287.

## Supporting information

This article contains supporting information.

## Conflicts of interest

The authors declare no conflicts of interest with the contents of this article.

## References

[1] Fialkow P.J., Jacobson R.J., Papayannopoulou T. (1977). Chronic myelocytic leukemia: clonal origin in a stem cell common to the granulocyte, erythrocyte, platelet and monocyte/macrophage. Am. J. Med..

[2] Jabbour E., Kantarjian H. (2012). Chronic myeloid leukemia: 2012 update on diagnosis, monitoring, and management. Am. J. Hematol..

[3] Kalidas M., Kantarjian H., Talpaz M. (2001). Chronic myelogenous leukemia. JAMA.

[4] Braun T.P., Eide C.A., Druker B.J. (2020). Response and resistance to BCR-ABL1-targeted therapies. Cancer Cell.

[5] Deininger M. (2011). Curing CML with imatinib—a dream come true?. Nat. Rev. Clin. Oncol..

[6] Apperley J.F. (2007). Part I: mechanisms of resistance to imatinib in chronic myeloid leukaemia. Lancet Oncol..

[7] Stege A., Priebsch A., Nieth C., Lage H. (2004). Stable and complete overcoming of MDR1/P-glycoprotein-mediated multidrug resistance in human gastric carcinoma cells by RNA interference. Cancer Gene Ther..

[8] Kuwazuru Y., Yoshimura A., Hanada S., Ichikawa M., Saito T., Uozumi K. (1990). Expression of the multidrug transporter, P-glycoprotein, in chronic myelogenous leukaemia cells in blast crisis. Br. J. Haematol..

[9] Herweijer H., Sonneveld P., Baas F., Nooter K. (1990). Expression of mdr1 and mdr3 multidrug-resistance genes in human acute and chronic leukemias and association with stimulation of drug accumulation by cyclosporine. J. Natl. Cancer Inst..

[10] Sharom F.J. (2008). ABC multidrug transporters: structure, function and role in chemoresistance. Pharmacogenomics.

[11] Ogretmen B., Safa A.R. (1999). Negative regulation of MDR1 promoter activity in MCF-7, but not in multidrug resistant MCF-7/Adr, cells by cross-coupled NF-κB/p65 and c-Fos transcription factors and their interaction with the CAAT region. Biochemistry.

[12] Bentires-Alj M., Barbu V., Fillet M., Chariot A., Relic B., Jacobs N. (2003). NF-κB transcription factor induces drug resistance through MDR1 expression in cancer cells. Oncogene.

[13] Hartmann G., Kim H., Piquette-Miller M. (2001). Regulation of the hepatic multidrug resistance gene expression by endotoxin and inflammatory cytokines in mice. Int. Immunopharmacol..

[14] Bauer B., Hartz A.M.S., Miller D.S. (2007). Tumor necrosis factor α and endothelin-1 increase P-glycoprotein expression and transport activity at the blood-brain barrier. Mol. Pharmacol..

[15] Heemskerk S., Peters J.G.P., Louisse J., Sagar S., Russel F.G.M., Masereeuw R. (2010). Regulation of P-glycoprotein in renal proximal tubule epithelial cells by LPS and TNF-α. J. Biomed. Biotechnol..

[16] Wang X., Huang S., Jiang Y., Liu Y., Song T., Li D. (2018). Reactive astrocytes increase the expression of P-gp and Mrp1 via TNF-α and NF-κB signaling. Mol. Med. Rep..

[17] Berguetti T., Quintaes L.S.P., Hancio T., Robaina M.C., Cruz A.L.S., Maia R.C. (2019). TNF-α modulates P-glycoprotein expression and contributes to cellular proliferation via extracellular vesicles. Cells.

[18] Thévenod F., Friedmann J.M., Katsen A.D., Hauser I.A. (2000). Up-regulation of multidrug resistance P-glycoprotein via nuclear factor-κB activation protects kidney proximal tubule cells from cadmium- and reactive oxygen species-induced apoptosis. J. Biol. Chem..

[19] Gasparini C., Celeghini C., Monasta L., Zauli G. (2014). NF-κB pathways in hematological malignancies. Cell. Mol. Life Sci..

[20] Han L., Zhang D., Tao T., Sun X., Liu X., Zhu G. (2015). The role of N-Glycan modification of TNFR1 in inflammatory microglia activation. Glycoconj. J..

[21] Holdbrooks A.T., Britain C.M., Bellis S.L. (2018). ST6Gal-I sialyltransferase promotes tumor necrosis factor (TNF)-mediated cancer cell survival via sialylation of the TNF receptor 1 (TNFR1) death receptor. J. Biol. Chem..

[22] Reily C., Stewart T.J., Renfrow M.B., Novak J. (2019). Glycosylation in health and disease. Nat. Rev. Nephrol..

[23] Ohtsubo K., Marth J.D. (2006). Glycosylation in cellular mechanisms of health and disease. Cell.

[24] Ferreira J.A., Peixoto A., Neves M., Gaiteiro C., Reis C.A., Assaraf Y.G. (2016). Mechanisms of cisplatin resistance and targeting of cancer stem cells: adding glycosylation to the equation. Drug Resist. Updat..

[25] Zhang Z., Zhao Y., Jiang L., Miao X., Zhou H., Jia L. (2012). Glycomic alterations are associated with multidrug resistance in human leukemia. Int. J. Biochem. Cell Biol..

[26] Li K., Sun Z., Zheng J., Lu Y., Bian Y., Ye M. (2013). In-depth research of multidrug resistance related cell surface glycoproteome in gastric cancer. J. Proteomics.

[27] Zeng W., Zheng S., Mao Y., Wang S., Zhong Y., Cao W. (2021). Elevated N-glycosylation contributes to the cisplatin resistance of non-small cell lung cancer cells revealed by membrane proteomic and glycoproteomic analysis. Front. Pharmacol..

[28] Cheng L., Luo S., Jin C., Ma H., Zhou H., Jia L. (2013). FUT family mediates the multidrug resistance of human hepatocellular carcinoma via the PI3K/Akt signaling pathway. Cell Death Dis..

[29] Kizuka Y., Taniguchi N. (2016). Enzymes for N-glycan branching and their genetic and nongenetic regulation in cancer. Biomolecules.

[30] Kudo T., Nakagawa H., Takahashi M., Hamaguchi J., Kamiyama N., Yokoo H. (2007). N-glycan alterations are associated with drug resistance in human hepatocellular carcinoma. Mol. Cancer.

[31] Guo R., Cheng L., Zhao Y., Zhang J., Liu C., Zhou H. (2013). Glycogenes mediate the invasive properties and chemosensitivity of human hepatocarcinoma cells. Int. J. Biochem. Cell Biol..

[32] Ma H., Miao X., Ma Q., Zheng W., Zhou H., Jia L. (2013). Functional roles of glycogene and N-glycan in multidrug resistance of human breast cancer cells. IUBMB Life.

[33] Lin G., Zhao R., Wang Y., Han J., Gu Y., Pan Y. (2020). Dynamic analysis of N-glycomic and transcriptomic changes in the development of ovarian cancer cell line A2780 to its three cisplatin-resistant variants. Ann. Transl. Med..

[34] Allam H., Johnson B.P., Zhang M., Lu Z., Cannon M.J., Abbott K.L. (2017). The glycosyltransferase GnT-III activates notch signaling and drives stem cell expansion to promote the growth and invasion of ovarian cancer. J. Biol. Chem..

[35] Taniguchi N., Ohkawa Y., Maeda K., Harada Y., Nagae M., Kizuka Y. (2020). True significance of N-acetylglucosaminyltransferases GnT-III, V and α1,6 fucosyltransferase in epithelial-mesenchymal transition and cancer. Mol. aspects Med..

[36] Schachter H. (1986). Biosynthetic controls that determine the branching and microheterogeneity of protein-bound oligosaccharides. Biochem. Cell Biol..

[37] Isaji T., Kariya Y., Xu Q., Fukuda T., Taniguchi N., Gu J., Fukuda M. (2010). Methods in Enzymology.

[38] Zhao R., Qin W., Qin R., Han J., Li C., Wang Y. (2017). Lectin array and glycogene expression analyses of ovarian cancer cell line A2780 and its cisplatin-resistant derivate cell line A2780-cp. Clin. Proteomics.

[39] da Fonseca L.M., Calvalhan D.M., Previato J.O., Mendonça Previato L., Freire-de-Lima L. (2020). Resistance to paclitaxel induces glycophenotype changes and mesenchymal-to-epithelial transition activation in the human prostate cancer cell line PC-3. Tumour Biol..

[40] Yoshimura M., Ihara Y., Taniguchi N. (1995). Changes of beta-1,4-N-acetylglucosaminyltransferase III (GnT-III) in patients with leukaemia. Glycoconj. J..

[41] Yoshimura M., Nishikawa A., Ihara Y., Nishiura T., Nakao H., Kanayama Y. (1995). High expression of UDP-N-acetylglucosamine: beta-D mannoside beta-1,4-N-acetylglucosaminyltransferase III (GnT-III) in chronic myelogenous leukemia in blast crisis. Int. J. Cancer.

[42] Vallabhapurapu S., Karin M. (2009). Regulation and function of NF-kappaB transcription factors in the immune system. Annu. Rev. Immunol..

[43] Horie R., Watanabe T. (1998). CD30: expression and function in health and disease. Semin. Immunol..

[44] Rebbaa A., Chou P.M., Vucic I., Mirkin B.L., Tomita T., Bremer E.G. (1999). Expression of bisecting GlcNAc in pediatric brain tumors and its association with tumor cell response to vinblastine. Clin. Cancer Res..

[45] Jiang L., Wang P., Sun Y.J., Wu Y.J. (2019). Ivermectin reverses the drug resistance in cancer cells through EGFR/ERK/Akt/NF-κB pathway. J. Exp. Clin. Cancer Res..

[46] Takada Y., Kobayashi Y., Aggarwal B.B. (2005). Evodiamine abolishes constitutive and inducible NF-κB activation by inhibiting IκBα kinase activation, thereby suppressing NF-κB-regulated antiapoptotic and metastatic gene expression, up-regulating apoptosis, and inhibiting invasion. J. Biol. Chem..

[47] Wang W., McLeod H.L., Cassidy J. (2003). Disulfiram-mediated inhibition of NF-κB activity enhances cytotoxicity of 5-fluorouracil in human colorectal cancer cell lines. Int. J. Cancer.

[48] Yu H., Lin L., Zhang Z., Zhang H., Hu H. (2020). Targeting NF-κB pathway for the therapy of diseases: mechanism and clinical study. Signal Transduct. Target. Ther..

[49] Sprowl J.A., Reed K., Armstrong S.R., Lanner C., Guo B., Kalatskaya I. (2012). Alterations in tumor necrosis factor signaling pathways are associated with cytotoxicity and resistance to taxanes: a study in isogenic resistant tumor cells. Breast Cancer Res..

[50] Chan F.K.-M. (2007). Three is better than one: pre-ligand receptor assembly in the regulation of TNF receptor signaling. Cytokine.

[51] Yoshimura M., Ihara Y., Ohnishi A., Ijuhin N., Nishiura T., Kanakura Y. (1996). Bisecting N-acetylglucosamine on K562 cells suppresses natural killer cytotoxicity and promotes spleen colonization. Cancer Res..

[52] Isaji T., Sato Y., Fukuda T., Gu J. (2009). N-glycosylation of the I-like domain of β1 integrin is essential for β1 integrin expression and biological function: identification OF the MINIMAL N-glycosylation REQUIREMENT for α5β1∗. J. Biol. Chem..

[53] Qi F., Isaji T., Duan C., Yang J., Wang Y., Fukuda T. (2020). ST3GAL3, ST3GAL4, and ST3GAL6 differ in their regulation of biological functions via the specificities for the α2,3-sialylation of target proteins. FASEB J..

[54] Sasai K., Ikeda Y., Ihara H., Honke K., Taniguchi N. (2003). Caveolin-1 regulates the functional localization of N-acetylglucosaminyltransferase III within the Golgi apparatus. J. Biol. Chem..

[55] Osuka R.F., Hirata T., Nagae M., Nakano M., Shibata H., Okamoto R. (2022). N-Acetylglucosaminyltransferase-V requires a specific non-catalytic luminal domain for its activity toward glycoprotein substrates. J. Biol. Chem..

[56] Shimizu S. (2019). Organelle zones in mitochondria. J. Biochem..

[57] Kurokawa K., Osakada H., Kojidani T., Waga M., Suda Y., Asakawa H. (2019). Visualization of secretory cargo transport within the Golgi apparatus. J. Cell Biol..

[58] Sasaki K., Yoshida H. (2019). Organelle zones. Cell Struct. Funct..

[59] Yano H., Yamamoto-Hino M., Abe M., Kuwahara R., Haraguchi S., Kusaka I. (2005). Distinct functional units of the golgi complex in drosophila cells. Proc. Natl. Acad. Sci. U. S. A..

[60] Iijima J., Zhao Y., Isaji T., Kameyama A., Nakaya S., Wang X. (2006). Cell-cell interaction-dependent regulation of N-acetylglucosaminyltransferase III and the bisected N-glycans in GE11 epithelial cells. Involvement of E-cadherin-mediated cell adhesion. J. Biol. Chem..

[61] Akama R., Sato Y., Kariya Y., Isaji T., Fukuda T., Lu L. (2008). N-acetylglucosaminyltransferase III expression is regulated by cell-cell adhesion via the E-cadherin–catenin–actin complex. Proteomics.

[62] Xu Q., Akama R., Isaji T., Lu Y., Hashimoto H., Kariya Y. (2011). Wnt/β-Catenin signaling down-regulates N-acetylglucosaminyltransferase III expression: the implications of two mutually exclusive pathways for regulation. J. Biol. Chem..

[63] Martin-Orozco E., Sanchez-Fernandez A., Ortiz-Parra I., Ayala-San Nicolas M. (2019). WNT signaling in tumors: the way to evade drugs and Immunity. Front. Immunol..

[64] Anugraham M., Jacob F., Nixdorf S., Everest-Dass A.V., Heinzelmann-Schwarz V., Packer N.H. (2014). Specific glycosylation of membrane proteins in epithelial ovarian cancer cell lines: glycan structures reflect gene expression and DNA methylation status. Mol. Cell. Proteomics.

[65] Vojta A., Samaržija I., Bočkor L., Zoldoš V. (2016). Glyco-genes change expression in cancer through aberrant methylation. Biochim. Biophys. Acta.

[66] Isaji T., Im S., Gu W., Wang Y., Hang Q., Lu J. (2014). An oncogenic protein golgi phosphoprotein 3 up-regulates cell migration via sialylation. J. Biol. Chem..

[67] Sato Y., Isaji T., Tajiri M., Yoshida-Yamamoto S., Yoshinaka T., Somehara T. (2009). An N-glycosylation site on the beta-propeller domain of the integrin alpha5 subunit plays key roles in both its function and site-specific modification by beta1,4-N-acetylglucosaminyltransferase III. J. Biol. Chem..

[68] Hang Q., Isaji T., Hou S., Im S., Fukuda T., Gu J. (2015). Integrin α5 suppresses the Phosphorylation of epidermal growth factor receptor and its cellular signaling of cell proliferation via N-glycosylation. J. Biol. Chem..

[69] Nakano M., Saldanha R., Göbel A., Kavallaris M., Packer N.H. (2011). Identification of glycan structure alterations on cell membrane proteins in desoxyepothilone B resistant leukemia cells. Mol. Cell. Proteomics.

[70] Nakano M., Mishra S.K., Tokoro Y., Sato K., Nakajima K., Yamaguchi Y. (2019). Bisecting GlcNAc is a general suppressor of terminal modification of N-glycan. Mol. Cell. Proteomics.

[71] Tomida S., Takata M., Hirata T., Nagae M., Nakano M., Kizuka Y. (2020). The SH3 domain in the fucosyltransferase FUT8 controls FUT8 activity and localization and is essential for core fucosylation. J. Biol. Chem..

[72] Duan C., Fukuda T., Isaji T., Qi F., Yang J., Wang Y. (2020). Deficiency of core fucosylation activates cellular signaling dependent on FLT3 expression in a Ba/F3 cell system. FASEB J..

