# Supplementary material for: Expression of GnT-III decreases chemoresistance via negatively regulating P-glycoprotein expression: Involvement of the TNFR2-NF-κB signaling pathway
Source: J Biol Chem. 2023 Feb 21;299(4):103051. doi: 10.1016/j.jbc.2023.103051 (PMC10033316; doi:10.1016/j.jbc.2023.103051)
Supplement: Supporting Table S1 [file mmc1.pdf]

Supplementary Table 1

LC-ESI MS analysis of *N*-glycans in K562 and K562/ADR cells after desialylation

Structure		Relative Abundances (%)	
Composition	Number	K562 desialo	K562/ADR desialo
(Hex)5 + (Man)3(GlcNAc)2	3	6.96	8.49
(Hex)2 (HexNAc)2 + (Man)3(GlcNAc)2	17	2.28	5.90
(Hex)3 (HexNAc)3 + (Man)3(GlcNAc)2	29	1.84	3.08
(Hex)4 (HexNAc)4 + (Man)3(GlcNAc)2	40	0.66	1.40
(Hex)2 (HexNAc)2 (Deoxyhexose)1 + (Man)3(GlcNAc)2	18	9.43	17.47
(Hex)3 (HexNAc)3 (Deoxyhexose)1 + (Man)3(GlcNAc)2	31	5.56	4.98
(Hex)4 (HexNAc)4 (Deoxyhexose)1 + (Man)3(GlcNAc)2	41	6.09	6.17
(Hex)2 (HexNAc)3 (Deoxyhexose)1 + (Man)3(GlcNAc)2	28	6.14	1.10
(Hex)3 (HexNAc)4 (Deoxyhexose)1 + (Man)3(GlcNAc)2	38	4.22	0.53
(Hex)4 (HexNAc)5 (Deoxyhexose)1 + (Man)3(GlcNAc)2	49	9.58	1.22
(Hex)2 (HexNAc)3 + (Man)3(GlcNAc)2	27	1.03	0.42
(Hex)3 (HexNAc)4 + (Man)3(GlcNAc)2	37	0.66	0.30
(Hex)4 (HexNAc)5 + (Man)3(GlcNAc)2	48	0.67	0.18
	SUM	55.14	51.24
